# Water Content of Polyelectrolyte Multilayer Films Measured by Quartz Crystal Microbalance and Deuterium Oxide Exchange

**DOI:** 10.3390/s21030771

**Published:** 2021-01-24

**Authors:** Joshua Kittle, Jacob Levin, Nestor Levin

**Affiliations:** Department of Chemistry, United States Air Force Academy, 2355 Fairchild Drive, Colorado Springs, CO 80840, USA; C21Jacob.Levin@afacademy.af.edu (J.L.); C22Nestor.Levin@afacademy.af.edu (N.L.)

**Keywords:** water content, thin film, quartz crystal microbalance, polyelectrolyte, deuterated solvent, PAH, PSS

## Abstract

Water content of natural and synthetic, thin, polymer films is of considerable interest to a variety of fields because it governs properties such as ion conductivity, rigidity, porosity, and mechanical strength. Measuring thin film water content typically requires either complicated and expensive instrumentation or use of multiple instrumental techniques. However, because a quartz crystal microbalance (QCM) is sensitive to changes in mass and viscosity, deuterated solvent exchange has emerged as a simple, single-instrument, in situ method to quantify thin film water content. Relatively few studies, though, have employed this technique to measure water content of polyelectrolyte multilayers formed by layer-by-layer (LbL) assembly. In this work, poly (allyl amine) (PAH) and poly (styrene sulfonate) (PSS) films of up to nine layers were formed and the water content for each layer was measured via QCM with deuterium oxide exchange. The well-characterized nature of PAH/PSS films facilitated comparisons of the technique used in this work to other instrumental methods. Water content results showed good agreement with the literature and good precision for hydrated films thicker than 20 nm. Collectively, this work highlights the utility, repeatability, and limitations of this deuterated exchange technique in measuring the solvent content of thin films.

## 1. Introduction

Determining the water content within polymer thin films remains an important parameter for a number of disciplines. For example, ionomer water content affects ion conductivity and, thus, the efficiency of these materials in proton exchange membrane fuel cells [1,2,3]. Likewise, the hydration level of thin films from natural materials, such as cellulose or lipid bilayers, is important for mimicking the mechanical properties of biological systems [4,5,6,7,8]. Measuring water uptake is also important for characterizing polymers used as protective coatings or for promoting flocculation [9,10,11,12]. While a number of methods exist to measure water content of thin films, these methods generally require either unique facilities (e.g., neutron reflectivity) or multiple instrumental techniques. 

Outside of neutron reflectivity, the most common method to measure water content of thin films is by quartz crystal microbalance (QCM) paired with an additional dry mass technique [13,14,15,16]. In brief, QCM drives a quartz crystal to its resonant frequency. As mass is added to the crystal surface, or the density, viscosity, or surface tension, or interfacial wetting of the surrounding medium changes, the resonant behavior of the quartz crystal changes [17,18,19,20]. Originally designed to measure the adsorption of gases to the crystal surface, the Sauerbrey equation relates the decrease in resonant frequency to the adsorbed mass [21]. For measurements in liquids or for the adsorption of floppy polymer layers, changes in viscoelasticity also contribute to decreases in crystal resonant frequency. By using a quartz crystal microbalance with dissipation monitoring (e.g., QCM-D), the contributions of density and viscosity of liquids or the polymer viscoelasticity of adsorbed layers can be modeled with the Kanazawa and Gordon equation or the Voigt model, respectively [22,23,24]. However, when changes in dissipation (Δ*D*) are less than 5% of the scaled frequency change, adsorbed masses are generally considered rigid and the Sauerbrey equation is valid [25]. Relevant to this work, QCM measures the wet mass of an adsorbed material to the crystal surface, e.g., a polymer film as well as any solvent entrapped or hydrodynamically associated with that film. Thus, to measure the water content of a film, QCM is usually paired with another technique that only measures the dry mass of polymer film. The mass difference between the two techniques permits calculation of the percentage of water within the film.

While several techniques have been used to measure thin film dry mass, surface plasmon resonance (SPR) and ellipsometry are most common. Though detailed descriptions of these two experimental techniques are outside the scope of this work, both methods are optically based and provide a dry mass of polymer film [26,27]. SPR and ellipsometry are typically used as standalone techniques when measuring thin film water content, meaning that to determine both the wet and dry mass, one set of experiments must be run with QCM, while a separate set of experiments under identical conditions must be run by either SPR or ellipsometry. However, with appropriate experimental design and a tailored QCM crystal sensor mount, SPR and ellipsometry can be paired with QCM for simultaneous, in situ measurement of both wet and dry mass of adsorbed films [13,14]. Regardless, determining thin film water content has generally required two instrumental methods and separate measurements on each instrument [8,15], increasing both the cost, complexity, and time associated with measuring this parameter.

QCM-D with deuterated solvent exchange is a single-instrument method that involves a two-step experiment: a standard thin-film adsorption experiment from solution that measures the wet mass, followed immediately by a switch from the solvent to the deuterated solvent [28,29]. The additional mass of the deuterated solvent is detected by the QCM-D and is related to the exchangeable solvent within the thin film. This difference in adsorbed wet mass and the adsorbed deuterated mass can be used to calculate the solvent content of the thin film using a single, in situ technique. Of note, this deuterated solvent method relies on a physical diffusion process for the exchange of solvent with deuterated solvent [28]. To prevent frequency shifts of indeterminate cause, the solvent and deuterated solvent should be paired (e.g., water and deuterium oxide). This method assumes that the chemical interactions of the solvent and deuterated solvent with the film are identical and, thus, solvent and deuterated solvent are exchangeable within the film [28]. Otherwise, significant differences in the viscosity of the solvent and deuterated solvent, as well as conformational changes in the thin film caused by differences in solvent-induced polymer swelling, would also contribute to the frequency shift [22,23,28].

QCM-D with deuterated solvent exchange has been used previously to determine thin film water content, primarily for model plant cell wall materials such as cellulose [29,30], xyloglucan [8,31], dextrins [32], and other natural polymers [6,33,34]. Craig et al. pioneered this technique and studied the water content of a single layer of a cationic random copolymer of an acrylamide and [3-(2-methylpropionamide)propyl] trimethyl ammonium [28]. While the results compared favorably to results from X-ray photoelectron spectroscopy, a layer-by-layer (LbL) film was not studied and the repeatability of the result was not reported. Notley et al. used this technique to study water contents of a polyelectrolyte film with up to four layers of polyallylamine hydrochloride (PAH) and polyacrylic acid, finding a decrease in water content with increasing film thickness [35]. Again, though, the repeatability of the results was not reported.

In this work, frequency shifts measured by QCM-D upon deuterated solvent exchange were used to determine the water content of layer-by-layer (LbL) polyelectrolyte films of cationic PAH and anionic polystyrene sulfonate (PSS) assembled onto gold sensor crystals for films of up to nine layers. Though this single-instrumental method has been used to determine thin film water content previously, a detailed study of the accuracy and utility of this technique over multiple LbL polyelectrolyte film thicknesses for a well-characterized system has not been examined. A deuterium oxide exchange was performed after addition of each layer, enabling the calculation of the average thin film water content with standard deviation for each of the nine polyelectrolyte layers. The results indicated good agreement with literature studies that employed both QCM-D and ellipsometry to measure water content [14], though the accuracy and precision of the deuterated technique increased for thicker films with higher water content, highlighting the limitations of this method in measuring the water content of very thin films (<20 nm) with relatively little associated water.

## 2. Materials and Methods

Poly (sodium 4-styrenesulfonate) (PSS, 70 kDa), poly (allylamine hydrochloride) (PAH, 20 wt% in H_2_O, 17 kDa), deuterium oxide (D_2_O, 99.9%), and sodium chloride (NaCl, 99.5%) were purchased from Sigma Aldrich. Hydrogen peroxide (H_2_O_2_, 30 wt%) and ammonium hydroxide (NH_4_OH, certified ACS Plus) used for cleaning sensor crystals (Q-Sense AB, gold, 5 MHz) were purchased from Fisher Scientific. All reagents were used as received. Ultrapure water (Millipore Milli-Q Gradient A-10, resistivity 18 MΩ·cm, <5 ppb inorganic impurities) was used in all experiments.

An E-4 QCM-D (Q-Sense AB) was used to measure frequency shifts associated with deposition of polyelectrolyte layers and deuterated water exchange. Prior to use, sensor crystals were cleaned via UV/ozone for 20 min, followed by rinsing with ultrapure water and immersion into a 1:1:5 solution of H_2_O_2_/NH_4_OH/water at 80 °C for 1 h. After another cleaning with UV/ozone and subsequent drying with ultrapure nitrogen (Airgas), sensor crystals were mounted into the QCM-D. Additionally, sensor holders were regularly disassembled and the flow cell was placed into a 2 wt% solution of sodium dodecyl sulfate (SDS), heated to 60 °C, sonicated for 15 min, and dried with nitrogen. Regular cleaning of the flow cells was required due to the high salt content of the polyelectrolyte solutions. After detailed cleaning of the flow cells, the sensor holders were reassembled and flushed with ultrapure water before clean sensor crystals were mounted into the holders.

Layer-by-layer (LbL) polyelectrolyte films of alternating PAH and PSS layers were formed on the gold substrate of the sensor crystal by passing 1 mg/mL polyelectrolyte solutions in 0.5 M NaCl through the flow cell, following the procedure of Iturri Ramos et al. [14]. Initially, a 0.5 M NaCl solution was passed through the flow cell at a rate of 0.200 mL/min at 25 °C for about 1 hr until a stable frequency baseline was established. Then, the first polyelectrolyte layer was adsorbed to the gold substrate by flowing the PAH solution for 12 min at a rate of 0.200 mL/min, followed by a 12-min rinse with the 0.5 M NaCl solution at a rate of 0.200 mL/min. Likewise, the next polyelectrolyte layer was then adsorbed by introducing the PSS solution for 12 min, followed by a 12-min rinse with the 0.5 M NaCl solution. This process was repeated until the desired number of polyelectrolyte multilayers had been adsorbed to the surface. After the final rinse with 0.5 M NaCl and when the frequency was stable, D_2_O was introduced into the flow cell for 5 min at a rate of 0.200 mL/min, followed by a rinse with ultrapure water [29].

The frequency shift (Δ*f*) for the buildup of a polyelectrolyte film and its associated deuterated water exchange was recorded for the fifth overtone (*n* = 5) for each of the nine layers. Because the measured dissipation values (Δ*D*) for polyelectrolyte adsorption were less than 5% of the scaled frequency shift (Δ*f*/*n*), the Sauerbrey equation was assumed valid and the surface excess (Δ*m*, ng·cm^−2^) of the adsorbed mass could be calculated via [21,25]:

(1) $$\Delta m=-C{\left(\frac{\Delta f}{n}\right)}_{m}$$

where Δ*f* is the change in frequency caused by adsorption to the sensor substrate, *n* is the frequency overtone (*n* = 5 for this work), and *C* is the Sauerbrey constant (17.7 ng·s·cm^−2^). The film thickness (*d*_film_) was determined using the density of the hydrated polyelectrolyte film (*ρ_film_*) via:

(2) dfilm=Δmρfilm

where the value of *ρ_film_* was assumed to be essentially that of water (1.0 g·cm^-3^) according to previous LbL polyelectrolyte thin film studies [14].

The exchangeable water content of the polyelectrolyte film, (Δ*f*/*n*)*_film water_*, was determined from the measured deuterated solvent exchange data using a previously published technique [28,29]. For this method,

(3) $${\left(\frac{\Delta f}{n}\right)}_{film\;water}=-\frac{{\left(\frac{\Delta f}{n}\right)}_{D2O:film}-{\left(\frac{\Delta f}{n}\right)}_{D2O:bare}}{\begin{array}{c} \begin{array}{c} \frac{\rho_{D2O}}{\rho_{H2O}}-1 \end{array} \end{array}}$$

where $\rho_{D2O}$ is the density of deuterium oxide (1.044 g·cm^−3^ at 25 °C) and $\rho_{H2O}$ is the density of water (0.9970 g·cm^−3^ at 25 °C) [36,37]. The value (Δ*f*/*n*)*_D2O:bare_* is the scaled frequency shift for a bare gold QCM sensor crystal when the solvent is switched from H_2_O to D_2_O. This decrease in measured frequency arises from changes in the density and viscosity of D_2_O relative to H_2_O. Previous studies have measured this value to be −55.2 Hz for the fifth overtone (*n* = 5), in good agreement with the Kanazawa and Gordon equation [22]. The value (Δ*f*/*n*)*_D2O:film_* is the scaled frequency shift after a film has adsorbed to the QCM sensor surface and the solvent is switched from H_2_O to D_2_O. For a hydrated film, this frequency shift would be greater in magnitude than 55.2 Hz for the fifth overtone, as the value would include the shift expected for the change in density and viscosity of the solvent (e.g., 55.2 Hz) and the contribution of any exchangeable water within the film. Deuterium oxide exchanges with a scaled frequency shift with a magnitude less than 55.2 Hz for the fifth overtone have been reported for adsorbed polymer thin films and have been attributed to dewatering of the film [12].

The hydration of a film (%) can be calculated as the ratio of the exchangeable film water content, (Δ*f*/*n*)*_film water_*, and the frequency shift of the hydrated film, (Δ*f*/*n*)*_m_*, according to:

(4) $$hydration\;\left(\%\right)=\frac{{\left(\frac{\Delta f}{n}\right)}_{film\;water}}{{\left(\frac{\Delta f}{n}\right)}_{m}}\times 100\%$$

## 3. Results

**Figure 1:**
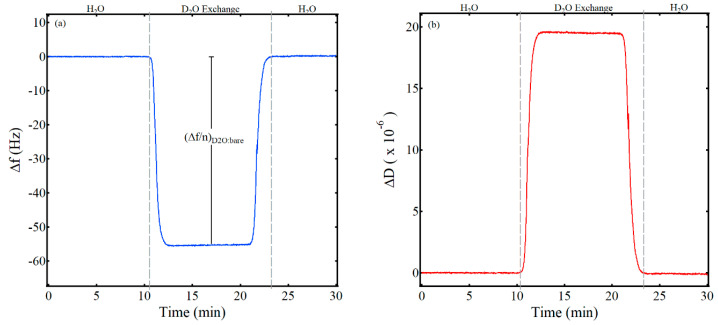
Frequency (**a**) and dissipation (**b**) values for a D_2_O exchange on a bare gold QCM sensor crystal. The vertical, gray, dashed lines highlight when the liquid was switched from water to D_2_O and back to water. The change in frequency for the deuterated solvent exchange of the bare crystal used to determine the thin film water content in Equation (3) is annotated as (Δ*f*/*n*)_*D2O:bare*_.

A key parameter in calculating the water content of a thin film via QCM and deuterated solvent exchange using Equation (3) is (Δ*f*/*n*)*_D2O:bare_*, the scaled frequency shift for a bare gold QCM sensor crystal when the solvent is switched from water to deuterium oxide. As noted in previous studies, the Kanazawa and Gordon equation predicts a shift of −55.9 Hz for the fifth overtone in switching from water to deuterated water and is based on the fundamental frequency of the crystal, the densities of the liquid and the quartz sensor crystal, the viscosity of the liquid, and the shear modulus of the quartz sensor crystal [22,29]. Figure 1 shows representative data for the experimentally determined frequency and dissipation shift in switching from water to deuterium oxide from this work. Initially, water flowed over the clean, bare sensor crystal at a flow rate of 0.200 mL/min and at a temperature of 25 °C for about 1 hr until a stable frequency baseline was established. Then, the liquid was switched from water to deuterium oxide, resulting in the significant decrease in frequency of about −55 Hz and labeled on Figure 1a as (Δ*f*/*n*)*_D2O:bare_*. As expected, dissipation also increased significantly due to the increase in the density and viscosity of deuterium oxide relative to water (Figure 1b). Both the frequency and dissipation returned to the baseline when the liquid was switched back to water. The results from three trials of this work yielded an average shift in frequency for the fifth overtone of (Δ*f*/*n*)*_D2O:bare_* = −55 Hz ± 1 Hz, in good agreement with previous work [29].

**Figure 2:**
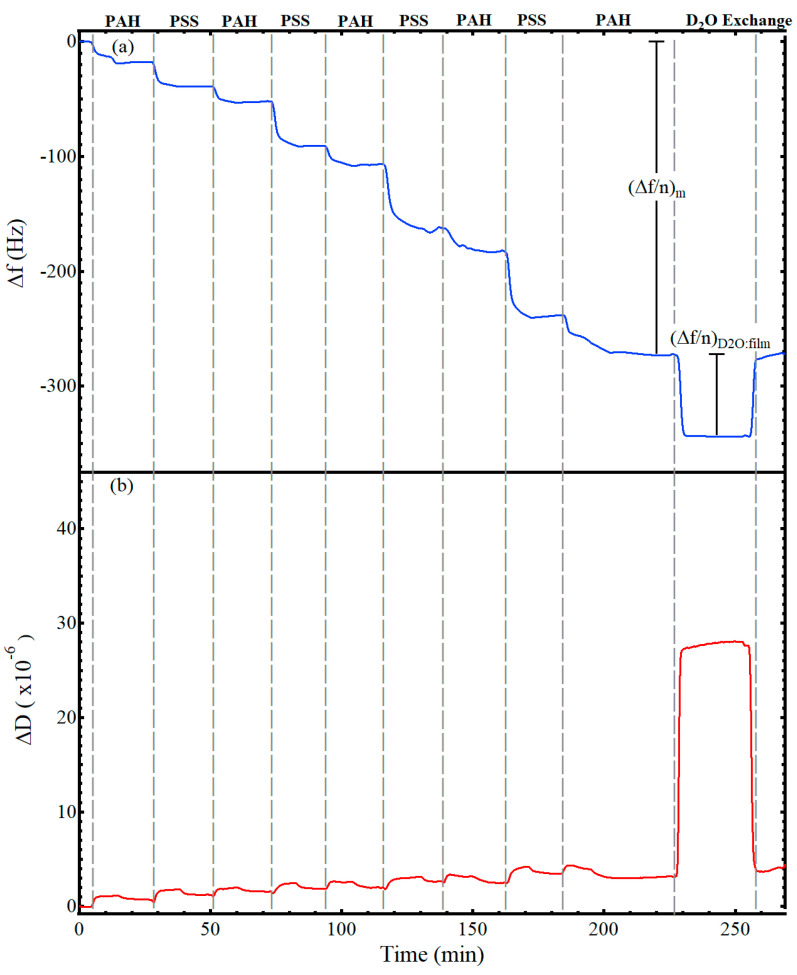
Frequency (**a**) and dissipation (**b**) values for the LbL adsorption of a nine-layer PAH/PSS polyelectrolyte film followed by D_2_O solvent exchange. The top of the figure annotates the polyelectrolyte layer adsorbed, while space between the vertical, dashed, gray lines include both the time for the polyelectrolyte adsorption and the associated salt rinse. The change in frequency used to calculate the hydrated surface excess Δm (Equation (1)) is annotated as (Δ*f*/*n*)_*m*_. The change in frequency for the deuterated solvent exchange of the adsorbed film used to determine the film water content Equation (3) is annotated as (Δ*f*/*n*)_*D2O:film*_.

Figure 2 shows representative data for the adsorption of a nine-layer, polyelectrolyte thin film from alternating solutions of 1 mg/mL PAH (0.5 M NaCl) and 1 mg/mL PSS (0.5 M NaCl). The gray, vertical lines indicate the switch to a different polyelectrolyte solution. While not annotated on the figure, after exposing the substrate to a polyelectrolyte solution for 12 min, the solution was switched to a salt rinse solution (0.5 M NaCl) for 12 min before introduction of the next polyelectrolyte solution, following the procedure of Iturri Ramos et al. [14]. Thus, a decrease in frequency was visible upon introduction of each polyelectrolyte solution as a hydrated layer adsorbed to the surface, followed by a flattening or slight increase upon introduction of the salt rinse, and then another frequency decrease with introduction of the oppositely charged polyelectrolyte. The frequency shift caused by the film and any associated water, (Δ*f*/*n*)*_m_*, is annotated on the figure. After forming the desired number of layers of polyelectrolytes, a deuterium oxide exchange was performed, resulting in the shift labeled as (Δ*f*/*n*)*_D2O:film_* on Figure 2a and used in Equation (3) to calculate the frequency shift associated with the hydrodynamically coupled water within the film. As visible in Figure 2a, the frequency decrease for the deuterium oxide exchange was closer to −65 Hz. Of this −65 Hz decrease, −55 Hz was attributed to the change in density and viscosity of the bulk solvent, e.g., Figure 1, and −10 Hz was attributed to the additional deuterium oxide that replaced the hydrodynamically coupled water within the film. The observed measurement drift of the E4 QCM-D was less than 0.5 Hz per hour and the noise level was less than 0.3 Hz for all experiments. Representative QCM-D results of LbL films with deuterium oxide solvent exchange for films with one to eight polyelectrolyte layers are provided in the Supplementary Materials Figures S1–S8.

**Figure 3:**
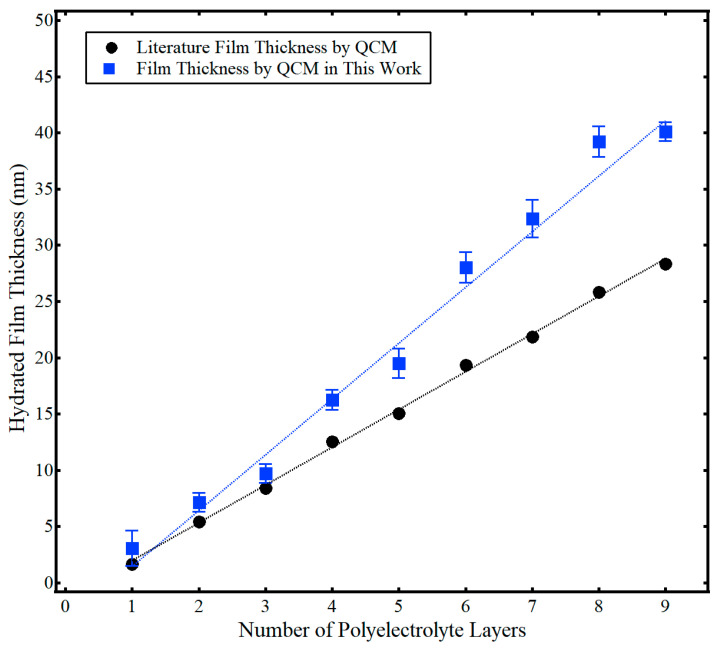
Hydrated film thickness (*d_film_*) as a function of the number of polyelectrolyte layers determined by QCM in this work (■) and results from Iturri Ramos et al. using QCM (●) [14]. Error bars indicate one standard deviation from the mean. Dashed lines represent a linear regression and are provided as a guide to the eye. Adapted from Jagoba J. Iturri Ramos, Stefan Stahl, Ralf P. Richter, and Sergio E. Moya *Macromolecules* **2010** *43* (21), 9063–9070. Copyright 2010 American Chemical Society.

The hydrated film thickness (*d_film_*) of the polyelectrolyte multilayer films is presented in Figure 3, along with associated literature results from Iturri Ramos et al. [14]. Again, this hydrated film thickness was calculated using Equation (2) via the total frequency shift of the hydrated polyelectrolyte film, (Δ*f*/*n*)*_m_*, from data similar to Figure 2. At least six trials of film thickness for each layer were collected over at least two separate measurement cycles. Both the work presented here and that from the literature showed a linear increase in the hydrated film thickness with the number of polyelectrolyte layers. However, the hydrated film thickness was larger in this work compared to Iturri Ramos et al. [14]. This difference likely stems from the use of different sensor crystal substrates. As discussed more fully in the subsequent section, Iturri Ramos et al. used a silica-coated sensor crystal [14], as opposed to the gold-coated sensor crystal used in this work. Regardless, the hydrated film thickness of the PAH/PSS LbL films of this work ranged in thickness from about 3 nm to 40 nm and was comparable to literature values [14]. Figure 3 also shows the cumulative standard deviation of the thickness of adsorbed polyelectrolyte with the addition of each layer. Formation of polyelectrolyte layers exhibited good repeatability, with a percent relative standard deviation generally less than 10%. Additional context can be found by examining the raw frequency data in the Supporting Information Figures S1–S8. Those raw data plots show frequency shifts for different sensors crystals performed on different measurement cycles. Thus, for the given experimental conditions (constant polyelectrolyte concentration, constant temperature), frequency shifts were repeatable within ~10%. For example, in comparing Figures S3–S8 for the addition of the third polyelectrolyte layer, the frequency decreased by ~50 Hz across all measurements. While there was more variation evident in the formation of the first two layers of polyelectrolyte, this likely stemmed from incomplete polyelectrolyte coverage of the sensor substrate for the initial PAH/PSS layers, as discussed more fully in the next section.

**Figure 4:**
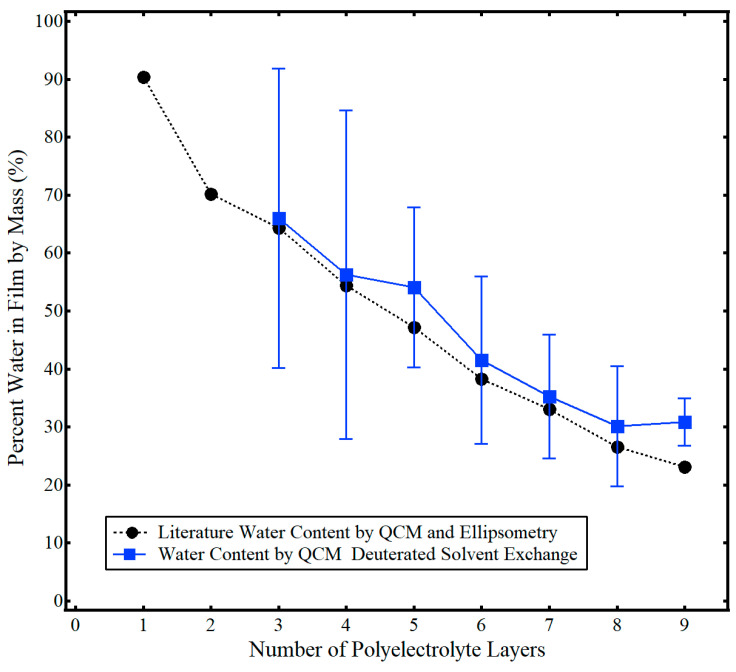
Percent water in PAH/PSS polyelectrolyte films as a function of the number of polyelectrolyte layers determined by QCM-D deuterated solvent exchange in this work (■) and results from Iturri Ramos et al. using QCM and ellipsometry (●) [14]. Error bars indicate one standard deviation from the mean. Adapted from Jagoba J. Iturri Ramos, Stefan Stahl, Ralf P. Richter, and Sergio E. Moya *Macromolecules* **2010** *43* (21), 9063–9070. Copyright 2010 American Chemical Society.

The percent water in the film by mass (e.g., hydration %) was calculated using Equations (3) and (4), data from Figure 1, and the averaged values for each polyelectrolyte layer with deuterated exchange similar to that found in Figure 2 and the Supporting Information (Figure 4). Generally, the average water content obtained by the single-instrument technique of this work matched that of the two-instrument method of Iturri Ramos et al. [14]. However, data for the first and second polyelectrolyte are not shown and the standard deviation error bars for the thinner polyelectrolyte layers three and four are relatively large (~30%). Six trials of solvent exchange data were collected for each layer over at least two separate measurement cycles. As noted previously in this work and similar to other studies, the repeatability of water–deuterium oxide exchange was measured to be ±2 Hz [27,28]. As discussed more fully in the next section, this result highlights a major limitation of the single-instrument deuterated solvent exchange method used to measure the solvent content of thin polymer films. In brief, very small shifts in measured deuterated solvent in a film, (Δ*f*/*n*)*_D2O:film_*, relative to that of a bare crystal, (Δ*f*/*n*)*_D2O:bare_*, lead to a 10-fold increase in calculated water content, e.g., the ratio of water and deuterium oxide densities in Equation (3). Thus, for very thin films in which (Δ*f*/*n*)*_D2O:film_* approaches the experimental repeatability of (Δ*f*/*n*)*_D2O:bare_* and the difference between (Δ*f*/*n*)*_D2O:film_* and(Δ*f*/*n*)*_D2O:bare_* is relatively small, significant error in measured solvent content arises.

## 4. Discussion

Thin films of PAH and PSS formed by LbL assembly are relatively well studied [14,38,39,40,41,42]. The thickness of PAH/PSS films adsorbed from aqueous salt solutions increases linearly, though the top-most layer of polyelectrolyte is known to affect the hydrated film thickness [38,39]. Generally, top-most layers of PSS have more hydrodynamically coupled water than do top-most layers of PAH, resulting in a zigzag growth in hydrated film thickness. As with most polyelectrolytes, PAH/PSS film thickness can be controlled by a variety of parameters, to include ionic strength of the polymer solution, rinsing protocols (e.g., water versus salt water) between polyelectrolyte depositions, temperature, pH, and underlying substrate [38,39,40,41,42]. Thicker films will typically form on a substrate when the polyelectrolyte in solution is in a more coiled, dense conformation [40]. For example, increasing ionic strength of a PAH solution screens the interchain electrostatic repulsion of a given PAH chain, resulting in coiling of the polyelectrolyte in solution and subsequent adsorption as thicker films [40]. Likewise, bulk polyelectrolyte adsorption to a neutral substrate (e.g., PAH to gold) versus an oppositely charged substrate (e.g., PAH to silica) yields thicker films as there are fewer electrostatic interactions with the surface to uncoil a given polyelectrolyte chain [41,42]. While the rinsing protocol between layers has been shown to affect the hydrated film thickness of PAH/PSS multilayers, the termination rinsing protocol (e.g., water or salt water) does not affect the final hydrated film thickness [40]. Evaluation of the water content of PAH/PSS multilayer films have shown that swelling of the film is most significant for the few top-most layers of polyelectrolyte because a rigid core of PAH electrostatically complexed with PSS, as well as hydrogen bonding between the PAH amine and the PSS oxygen, excludes exchangeable interior water [39,40]. Thus, the percent hydration of PAH/PSS films decreases with increasing polyelectrolyte layers: the water content of the top-most layers remains essentially constant but the overall mass of the film continues to increase.

The PAH/PSS assembly and water content measured in this work were in good agreement with the literature, highlighting the utility of QCM with deuterated solvent exchange as a single-instrument method to accurately measure percent hydration of a thin film. The characteristic zigzag increase in film thickness was evident in Figure 3 and the average thickness of a PAH/PSS bilayer was 10 nm ± 2 nm. This value was comparable but slightly higher than the values measured Iturri Ramos et al. [14]. This was attributed to the neutral gold substrate used in this work, rather than the charged silica substrate used in Iturri Ramos et al. [14], as neutral substrates have been shown to yield thicker polyelectrolyte films [42]. Average hydration of the film (Figure 4) was also in good agreement with the literature. As expected, the hydration decreased with increasing layers of PAH/PSS because of the exclusion of exchangeable water within the inner core of the polyelectrolyte film. For thicker polyelectrolyte layers (e.g., more than four layers), the exchangeable water within the film, (Δ*f*/*n*)*_film water_*, was essentially constant. This agreed with previously reported results, where the water content of the top-most layers of the polyelectrolyte was exchangeable but any water interior to the film was not [39,40]. Thus, the percent hydration of the entire film decreased with an increasing number of adsorbed polyelectrolyte layers.

While the method outlined here to determine the percent hydration of a thin polyelectrolyte film was generally successful, this study did highlight several limitations of this technique. As highlighted briefly in the Results section, this technique yielded unreasonable water content values for the first two polyelectrolyte layers (one layer: 260% ± 280%, two layer: 45% ± 63%). This was attributed to two factors. First, very small shifts in measured deuterated solvent in a film, (Δ*f*/*n*)*_D2O:film_*, relative to that of a bare crystal, (Δ*f*/*n*)*_D2O:bare_*, lead to a 10-fold increase in calculated water content, e.g., the ratio of water and deuterium oxide densities in Equation (3). Thus, for very thin films in which (Δ*f*/*n*)*_D2O:film_* approaches the experimental repeatability of (Δ*f*/*n*)*_D2O:bare_* (e.g., ±1 Hz) and the difference between (Δ*f*/*n*)*_D2O:film_* and(Δ*f*/*n*)*_D2O:bare_* is relatively small (2–3 Hz), significant error in water content arises. Second, the first few layers of polyelectrolytes assembled by the LbL technique are known to be non-uniform [43,44]. This inhomogeneity yields deviation in the initial quantity of polyelectrolyte and associated water adsorbed to the substrate. This deviation in both the film mass and water mass is propagated in subsequent calculations of the percent water. However, with the addition of subsequent polyelectrolyte layers and larger shifts in deuterated solvent exchange, the standard deviation of the measurements decreases, as shown in Figure 4, to values of less than 15% for layer five and less than 4% for layer nine. Thus, this water content technique for polyelectrolyte thin films matched the literature with good precision when hydrated film thicknesses were as low as 20 nm, with increasing precision for thicker films.

This QCM deuterated solvent exchange method offers advantages over more traditional methods to measure thin film water content, to include in situ measurement, a single instrument, and ease of use. While this technique has seen some use, primarily in measuring the water content of plant cell wall materials, this technique is well suited for expanded use in studying water content for a variety of materials [45,46,47,48]. Of particular interest would be the study of the water content of Nafion films by this method, as hydration of Nafion significantly affects the ion exchange of this potential fuel cell material [1]. Additionally, this deuterated solvent exchange method is not limited to studying film swelling by water and could be used to study swelling of polymers in other deuterated solvents, such as deuterated ethanol, benzene, or toluene [25].

## 5. Conclusions

This work highlights the advantages and limitations of the single-instrument method of QCM with deuterated solvent exchange to determine the solvent content of an adsorbed thin film. A relatively well-characterized system––the water content of thin LbL PAH/PSS multilayers––was used to evaluate this sensing method as a standalone technique for determining solvent content of thin films in greater detail than previous studies of polyelectrolyte systems. Results showed good agreement with the literature and good repeatability of the data, especially for thin films with thicknesses over 20 nm. Due to the simplicity of this method, reduced instrumentation requirements, and in situ measurements, this technique is well suited to measuring solvent content for a number of thin film polymer systems. 

## Data Availability

The data presented in this study are available on request from the corresponding author. Data were also obtained from Iturri Ramos et al. and are available from the authors at https://doi.org/10.1021/ma1015984 with the permission of the American Chemical Society.

## Supplementary Materials

The following are available online at https://www.mdpi.com/1424-8220/21/3/771/s1. Figure S1: Representative frequency shift data (*n* = 5) for the adsorption of a one-layer PAH film, followed by D_2_O solvent exchange. Figure S2: Representative frequency shift data (*n* = 5) for the adsorption of a two-layer PAH/PSS film, followed by D_2_O solvent exchange. Figure S3: Representative frequency shift data (*n* = 5) for the adsorption of a three-layer PAH/PSS film, followed by D_2_O solvent exchange. Figure S4: Representative frequency shift data (*n* = 5) for the adsorption of a four-layer PAH/PSS film, followed by D_2_O solvent exchange. Figure S5: Representative frequency shift data (*n* = 5) for the adsorption of a five-layer PAH/PSS film, followed by D_2_O solvent exchange. Figure S6: Representative frequency shift data (*n* = 5) for the adsorption of a six-layer PAH/PSS film, followed by D_2_O solvent exchange. Figure S7: Representative frequency shift data (*n* = 5) for the adsorption of a seven-layer PAH/PSS film, followed by D_2_O solvent exchange. Figure S8: Representative frequency shift data (*n* = 5) for the adsorption of an eight-layer PAH/PSS film, followed by D_2_O solvent exchange.
