# Supplementary material for: Water Content of Polyelectrolyte Multilayer Films Measured by Quartz Crystal Microbalance and Deuterium Oxide Exchange
Source: Sensors (Basel). 2021 Jan 24;21(3):771. doi: 10.3390/s21030771 (PMC7866239; doi:10.3390/s21030771)
Supplement: Supplementary file 1 [file sensors-21-00771-s001.pdf]

Supporting Information

Water Content of Polyelectrolyte Multilayer Films Measured by Quartz Crystal Microbalance and Deuterium Oxide Exchange

Joshua Kittle [1], Jacob Levin [1], Nestor Levin [1]*

[1] Department of Chemistry, United States Air Force Academy, 2355 Fairchild Drive, 80840 Colorado Springs, Colorado, United States; Joshua.Kittle@afacademy.af.edu (J.K.); C21Jacob.Levin@afacademy.af.edu (J.L.); C22Nestor.Levin@afacademy.af.edu (N.L.)

***To whom correspondences should be addressed:** Joshua.Kittle@afacademy.af.edu

Provided Supporting Information

I. Representative QCM Data for PAH/PSS Adsorption and D_2O Solvent Exchange

Figure S1: Representative frequency shift data (n = 5) for the adsorption of a one-layer PAH film, followed by D_2O solvent exchange.

Figure S2: Representative frequency shift data (n = 5) for the adsorption of a two-layer PAH/PSS film, followed by D_2O solvent exchange.

Figure S3: Representative frequency shift data (n = 5) for the adsorption of a three-layer PAH/PSS film, followed by D_2O solvent exchange.

Figure S4: Representative frequency shift data (n = 5) for the adsorption of a four-layer PAH/PSS film, followed by D_2O solvent exchange.

Figure S5: Representative frequency shift data (n = 5) for the adsorption of a five-layer PAH/PSS film, followed by D_2O solvent exchange.

Figure S6: Representative frequency shift data (n = 5) for the adsorption of a six-layer PAH/PSS film, followed by D_2O solvent exchange.

Figure S7: Representative frequency shift data (n = 5) for the adsorption of a seven-layer PAH/PSS film, followed by D_2O solvent exchange.

Figure S8: Representative frequency shift data (n = 5) for the adsorption of an eight-layer PAH/PSS film, followed by D_2O solvent exchange.

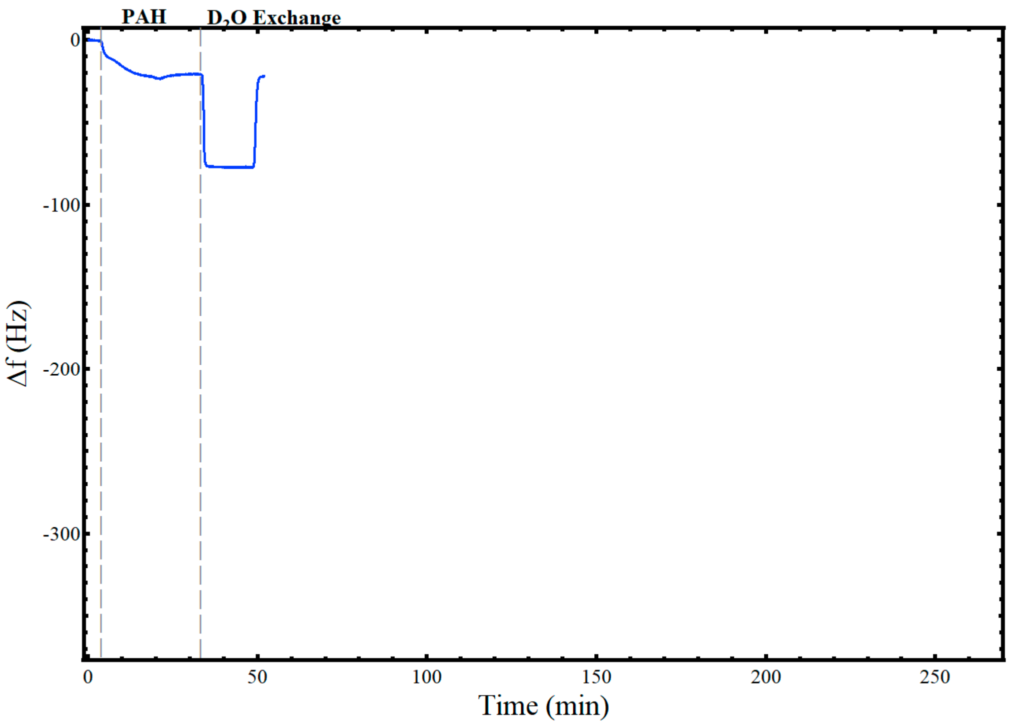

Figure S1: Representative frequency shift data (n = 5) for the adsorption of a one-layer PAH film, followed by D_2O solvent exchange.

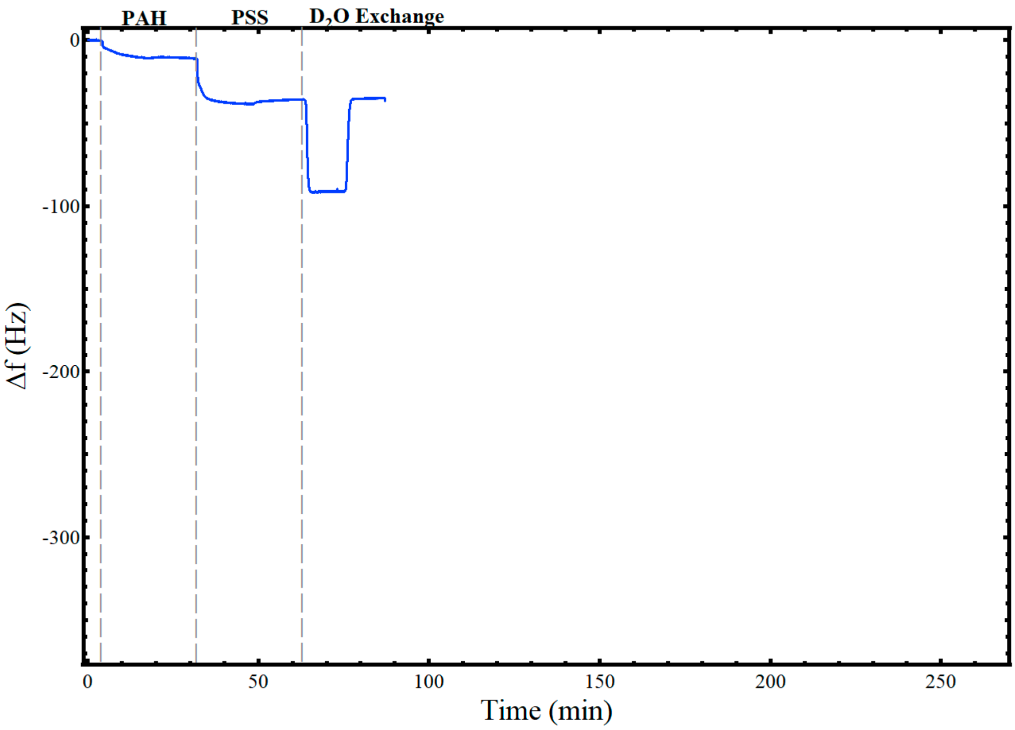

Figure S2: Representative frequency shift data (n = 5) for the adsorption of a two-layer PAH/PSS film, followed by D_2O solvent exchange.

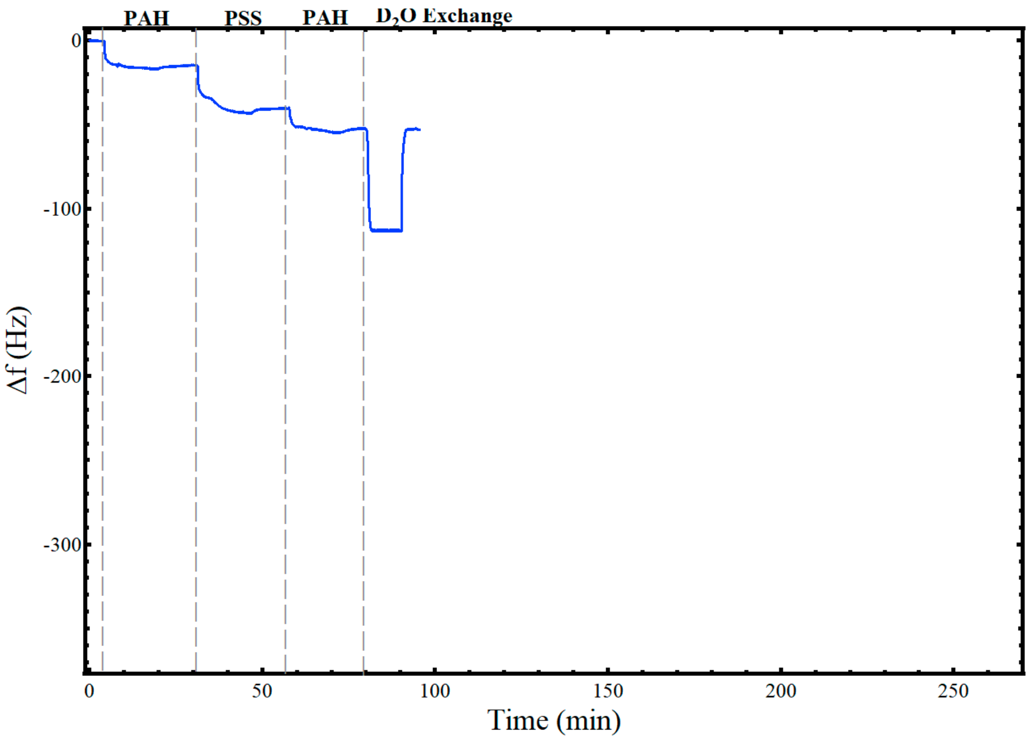

Figure S3: Representative frequency shift data (n = 5) for the adsorption of a three-layer PAH/PSS film, followed by D_2O solvent exchange.

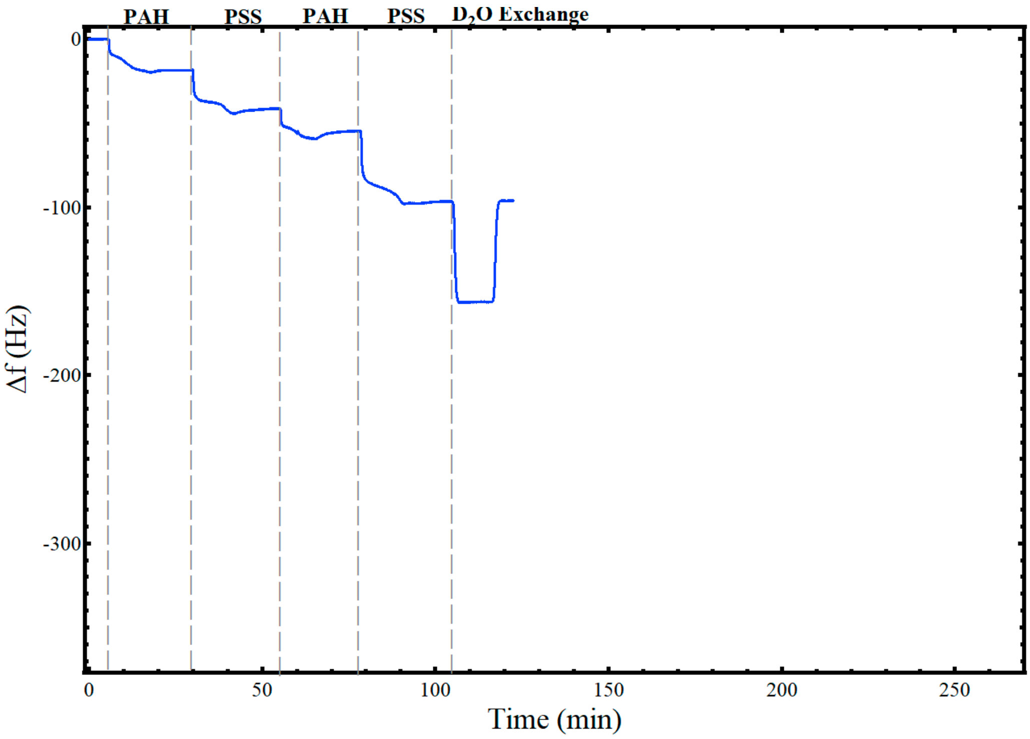

Figure S4: Representative frequency shift data (n = 5) for the adsorption of a four-layer PAH/PSS film, followed by D_2O solvent exchange.

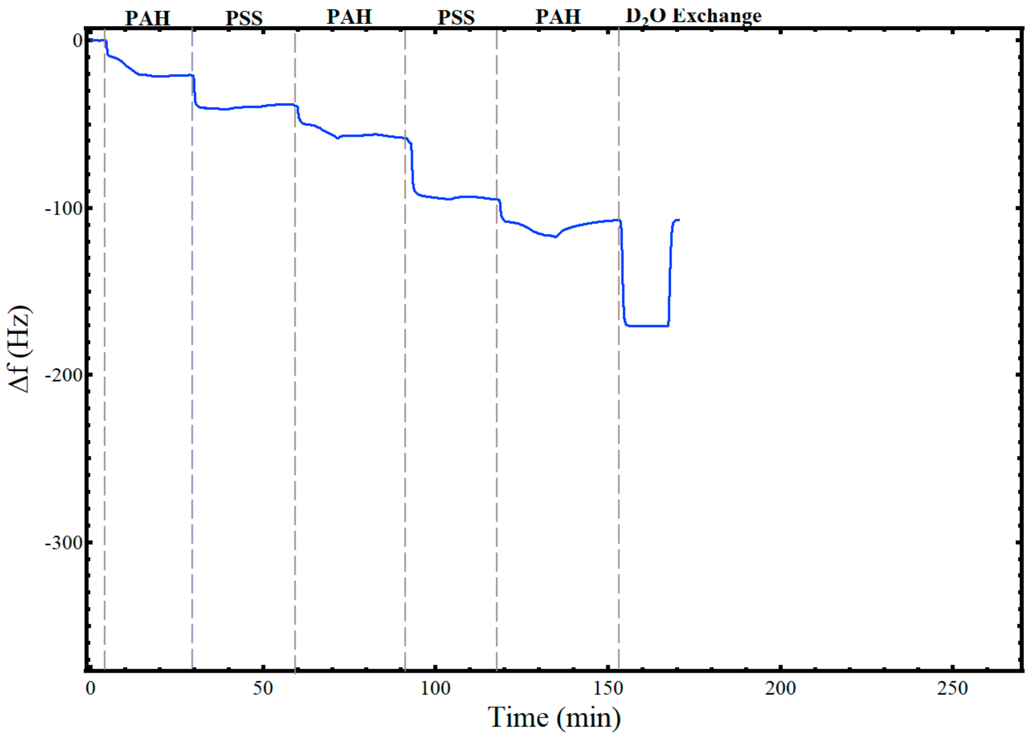

Figure S5: Representative frequency shift data (n = 5) for the adsorption of a five-layer PAH/PSS film, followed by D_2O solvent exchange.

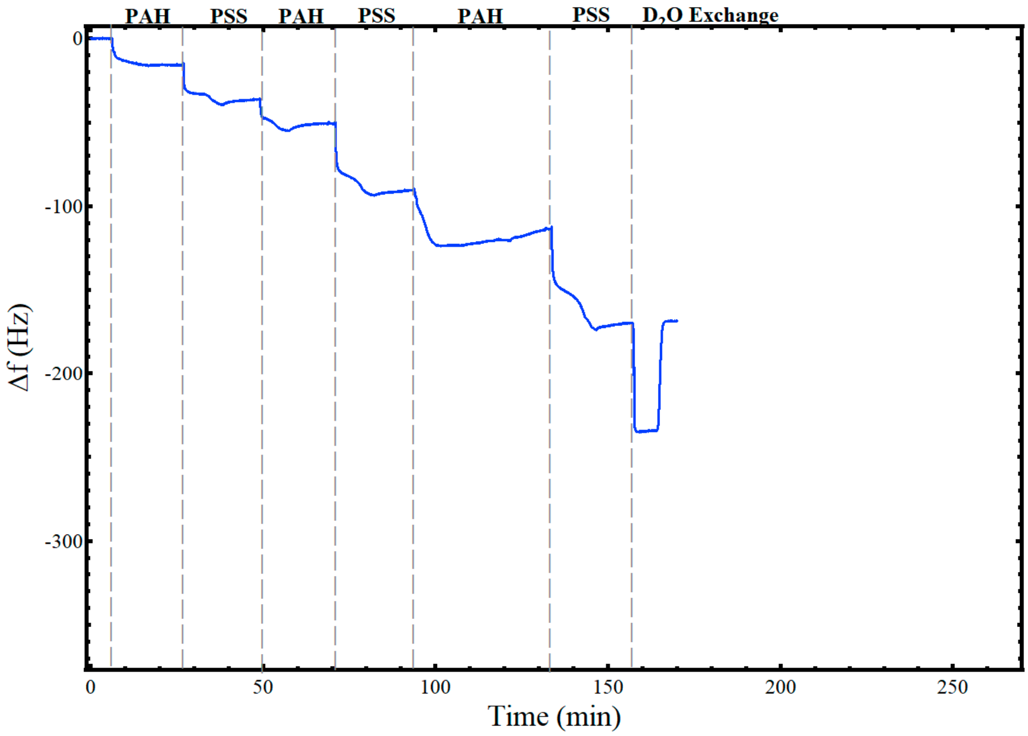

Figure S6: Representative frequency shift data (n = 5) for the adsorption of a six-layer PAH/PSS film, followed by D_2O solvent exchange.

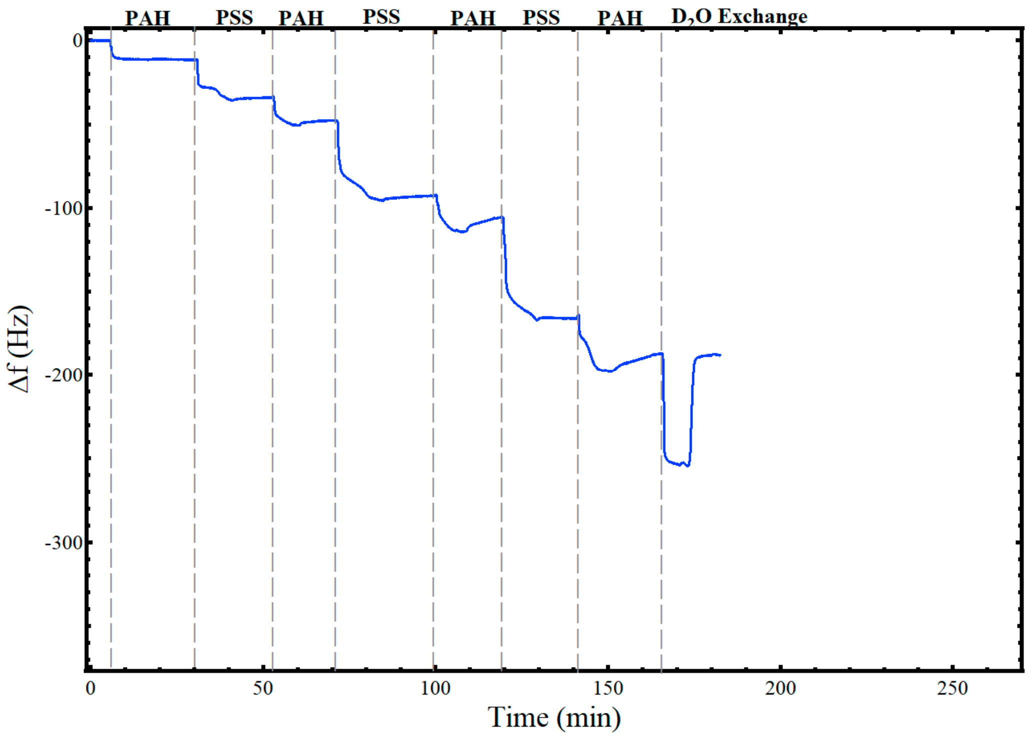

Figure S7: Representative frequency shift data (n = 5) for the adsorption of a seven-layer PAH/PSS film, followed by D_2O solvent exchange.

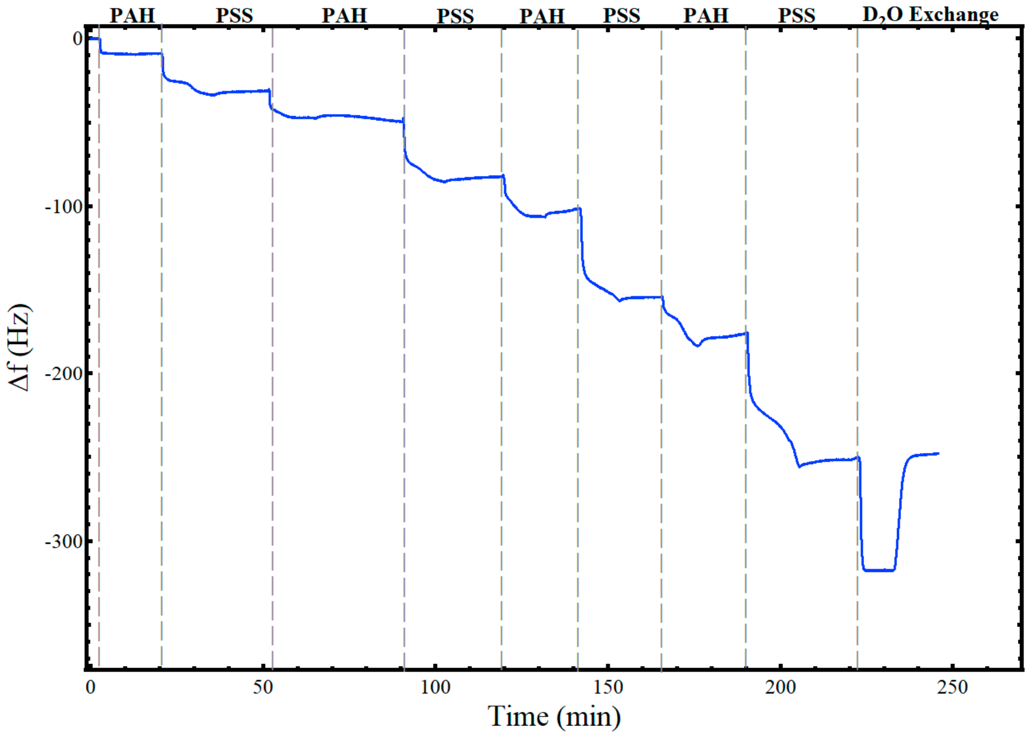

Figure S8: Representative frequency shift data (n = 5) for the adsorption of an eight-layer PAH/PSS film, followed by D_2O solvent exchange.
